# A shortened whole brain radiation therapy protocol for meningoencephalitis of unknown origin in dogs

**DOI:** 10.3389/fvets.2023.1132736

**Published:** 2023-03-20

**Authors:** Robert Herzig, Katrin Beckmann, Maximilian Körner, Frank Steffen, Carla Rohrer Bley

**Affiliations:** ^1^Division of Neurology, Department for Small Animals, Vetsuisse Faculty, University of Zurich, Zürich, Switzerland; ^2^Division of Radiation Oncology, Department for Small Animals, Vetsuisse Faculty, University of Zurich, Zürich, Switzerland

**Keywords:** MUO, MUE, encephalitis, inflammation, radiotherapy, canine, brain

## Abstract

**Introduction:**

A variety of treatment options have been described for canine meningoencephalitis of unknown origin (MUO). Few studies focused on radiation therapy as a second line immunomodulating treatment, implicating its effective use. However, a standard radiation therapy protocol is lacking, and further research will help to evaluate the effect of different dose regimens.

**Methods:**

Ten dogs diagnosed with MUO based on MRI and CSF findings were prospectively enrolled. The dogs were treated with a shortened whole brain radiation therapy protocol (5 × 4 Gy) in combination with prednisolone. Neurologic changes were quantified using an established scoring scheme. Follow-up MRI and CSF examination was scheduled three months after radiation therapy. Overall survival and time to progression were calculated. Histopathology of the brain was performed in case of death.

**Results:**

Seven dogs were diagnosed de novo and three had a history of relapsing MUO. Neurological status improved in all 10 dogs during radiation therapy, with 4/10 returning to normal shortly after radiation therapy. Three dogs died within the first three months after radiation therapy. At follow-up MRI lesions completely resolved in two dogs, partially resolved in five dogs, and progressed in one dog. After follow-up MRI, dogs were further treated with prednisolone monotherapy (two dogs) and additional immunosuppressant drugs (five dogs). Overall, four dogs showed disease progression, with a mean time to progression of 691 days (95%CI: 396–987) and mean overall survival for all dogs was 723 days (95%CI: 436–1011) (both medians not reached). Histopathology confirmed MUO in three dogs but was suggestive for oligodendroglioma in one dog. Radiation induced side effects were not seen.

**Conclusion:**

Shortened whole-brain radiation therapy could be an additional treatment option for MUO in conjunction to prednisolone, specifically for cases that require rapid relief of symptoms and with relapsing history.

## 1. Introduction

Meningoencephalitis of unknown origin (MUO) is a common central nervous system (CNS) disease in dogs and considered fatal if left untreated (1–3). While MUO can affect dogs of any breed, sex and age, small, toy and terrier breeds are most commonly affected (4). A large proportion, up to 56%, of affected dogs die or are euthanized, despite aggressive treatment (5). Regardless of the type of treatment, the mortality rate is highest within the first three months after diagnosis and ranges between 26 and 33% (3, 5–7). MUO is believed to be the result of an aberrant immune response against the CNS, but the exact pathomechanism is still incompletely understood (2, 8–10), which prohibits identifying more effective treatment strategies. In general, MUO-treatment bases on immunosuppressing the patient. Glucocorticoids, such as prednisolone are considered a cornerstone of therapy (4). A systematic review by Granger et al. indicated a possible benefit of a second-line immunosuppressive medication (11). Additionally, there is limited data that multi-drug immunomodulation may allow for faster dose-reduction of prednisolone (12), leading possibly to less glucocorticoid-associated side effects. In recent years, researchers have focused on the effect of second line immunosuppressants including azathioprine, cyclosporine, cytosine arabinoside, mycophenolate and others (2, 4). Yet, no preferred immunosuppressive strategy has been identified, mirroring the heterogenicity of the disease, as well as the varying inclusion criteria and drug regimens (4, 11). The remaining high mortality rate within the first months and various adverse effects of medical treatment highlight the need for continuous investigation in MUO treatment strategies.

So far, only three studies, investigated the effect of radiation therapy in conjunction to glucocorticoid treatment on MUO, with a total of 17 dogs (13–15). They demonstrated radiation therapy (RT) in addition to glucocorticoid treatment to be a feasible treatment option with rapid clinical improvement, including dogs refractory to medical therapy (15). However, a standard radiation therapy protocol is lacking as described protocols varied in delivered doses, fractions, and treatment times. Total doses between 30 and 49.5 Gy, divided into fractions of 2.4–4.0 Gy have been investigated.

Herein we wanted to evaluate a short, lower dose whole brain radiotherapy protocol in conjunction to prednisolone treatment in dogs diagnosed with focal or multifocal MUO. We delivered a total of 20 Gy in 4 Gy-fractions over five days. During follow-up, we investigated the outcome after treatment based on clinical-neurologic examinations, as well as MRI and CSF examinations.

## 2. Materials and methods

This prospective pilot study included 10 dogs with diagnosis of MUO. For the study, dogs were recruited between December 2019 and February 2022 at the Division of Neurology, Vetsuisse Faculty, University of Zurich. For all dogs, owners' informed consent was obtained for treatment and follow-up. Dogs were treated under approval of the Animal Ethics Council of the Canton of Zurich, Switzerland (Permit Number: ZH026/20).

### 2.1. Case selection

Clinical and neurologic examinations were performed by residency-trained or board-certified veterinary neurologist. In all dogs, complete blood cell count and serum biochemistry was performed.

Diagnosis of MUO was based on previously established inclusion criteria (11). The following inclusion criteria had to be fulfilled (1) evidence of focal or multifocal brain lesions during the neurological examination; (2) abnormal cerebrospinal fluid (CSF) (reference interval: <5 white blood cells (WBCs)/μL and/or total protein: <0.3 g/L, respective abnormal CSF cell differentiation) collected from the cerebromedullary cistern. In case the CSF total protein was determined by a Pandy test, a clear CSF was considered normal. Abnormal protein content was detected by turbidity and rated with maximum “+++”. In case CSF did not prove inflammation, diagnosis had to be confirmed by pathology; (3) relevant infectious diseases ruled out by testing from serum or CSF; (4) evidence of focal or multifocal intra-axial lesions in MRI, according to previously reported features (16–20).

MRI of the brain was performed with a high field scanner (1.5 or 3.0 T). Sequences included at least T2W images in transverse, sagittal and dorsal planes, T2W FLAIR in transverse plane and T1W transverse images acquired pre- and post-contrast medium application. MRIs were reported by a board-certified radiologist and reviewed by a board-certified neurologist.

Based on previously described MR imaging features of granulomatous meningoencephalitis (GME) (2, 16, 19), necrotizing leukoencephalitis (NLE) (2, 16, 21–23) and necrotizing meningoencephalitis (NME) (2, 16, 20, 22, 24) we grouped our cases into these subclassifications of MUO.

Dogs were not included if they presented with optic neuritis or spinal MUO only, or when their CSF analysis revealed eosinophilic or neutrophilic predominance.

Follow-up MRI was scheduled three months after the end of radiation therapy by a 3 Tesla MRI (Philips Ingenia scanner, Philips AG, 8027 Zurich, Switzerland). To allow for accurate comparison between the lesions prior and after radiation therapy, follow-up MRI included at least the same sequences.

### 2.2. Treatment

Whole-brain radiation therapy was delivered with photons of a 6 MV linear accelerator (Clinac iX, Varian, Palo Alto, California) equipped with a 5-mm leaf-width multileaf-collimator, using intensity-modulated radiation therapy (IMRT). Treatment planning was performed using Eclipse treatment planning software (Varian Oncology Systems, Palo Alto, California), applying AAA-algorithm (10.0.28). Radiation was planned isocentrically, with heterogeneity correction, by a board-certified radiation oncologist. Planning-CT and daily treatments were performed under general anesthesia in sternal recumbency. Reproducible positioning was accomplished with both, an individually shaped vacuum cushion and a custom-made bite block. The target was the whole brain, extended by a planning target volume of 2 mm (PTV) and dose was prescribed at the ICRU reference point and delivered in a protocol of 5 × 4 Gy (20 Gy total dose). According to the Swiss law and routine in our clinic, the IMRT treatment plans were dosimetrically verified using a phantom and a medical physicist approved all plans. All dogs received prednisolone, but no other immunosuppressive medication during radiation therapy.

### 2.3. Follow-up examinations and outcome

To quantify neurologic changes, the results of the neurologic examination were scored along a previously published scoring scheme (3) at the time of presentation, immediately before the first radiation therapy, after the end of radiation therapy and at the time of follow-up MRI and CSF examination. Progression free interval and overall survival from beginning of radiation therapy to time point of writing this study were documented.

### 2.4. Histopathology

Animals that died or have been euthanized underwent histopathologic examination of the brain to reach a definitive diagnosis, to determine the character and extend of inflammation and to assess the effect of radiation therapy on healthy CNS parenchyma. The examination was performed by a board-certified veterinary pathologist.

### 2.5. Statistical analysis

Descriptive statistics were used in the analysis of dogs and disease characteristics.

Time to progression was calculated from the first day of radiation therapy to the date of first-documented neurologic progression (clinical or imaging-based). Dogs showing no symptoms of deterioration, no progressive lesions on MRI or that were alive at the time of data analysis closure were censored. Dogs not progressing or alive at completion of data-analysis were censored. Overall survival was calculated from the first day of radiation therapy to the date of death. Dogs still alive at completion of data analysis were censored at last verified date alive as defined by follow-up exam or follow up phone call with the owners. All dogs that were dead at the end of the study were recorded as events. Survival plots were generated according to the Kaplan-Meier product-limit method. Survival estimates were presented as medians with the corresponding 95% confidence intervals (95% Cis).

## 3. Results

### 3.1. Study population

Ten dogs were enrolled prospectively in this study. Their signalment is given in Table 1. The mean age at diagnosis was 5.2 years (range 1.3–9).

**Table 1:** Signalment.

**Case no**.	**Dog breed**	**Sex**	**Age at time of diagnosis (months)**	**Relapse at inclusion**	**Relapse cases: age at relapse (months)**	**In case of relapse: previous treatment**		**Obtundation at presentation**	**Seizures**			**Infectious disease testing**	
						**Prednisolone**	**Other immunosuppressive drugs**		**At presentation**	**After RT**	**ASM**	**CSF**	**Blood**
1	French Bulldog	m	84	Yes	100	2 mg/kg q24h (1 week) 1 mg/kg q24h (6 weeks) 0.5 mg/kg q24h (11 weeks) 0.5 mg/kg q48h (6 weeks) 0.5 mg/kg q72h (7 weeks, then stopped) treatment stopped 37 weeks before relapse	Mycophenolate mofetil 18 mg/kg q48h (39 weeks, then stopped) treatment stopped 26 weeks before relapse	Yes	No	No	No	Neospora caninum (PCR) Distemper virus (PCR) TBE (Ab)	
2	Cocker Spaniel	m	39	Yes	65	0.5 mg/kg q24h (2 weeks) 1.3 mg/kg q24h (2 weeks) 0.5 mg/kg q24h (3 weeks) 0.3 mg/kg q24h (3 weeks) 0.15 mg/kg q24h (4 weeks) 0.15 mg/kg q48h (4 weeks, then stopped) Treatment stopped 11 weeks before first relapse 2 mg/kg q24h (2 weeks) 1.3 mg/kg q24h (2 weeks) 0.6 mg/kg q24h (2 weeks) 0.3 mg/kg q24h (8 weeks) 0.3 mg/kg q48h (12 weeks) 0.3 mg/kg q72h (11 weeks, then stopped) treatment stopped 46 weeks before second relapse	Ciclosporin (started after first relapse confirmed) 6.25 mg/kg q12h (4 weeks) 6.25 mg/kg q24h (42 weeks) 3.1 mg/kg q24h (26 weeks) Treated with 1.5 mg/kg for 16 weeks before second relapse	Yes	No	No	No	Toxoplasma gondii (PCR) Distemper virus (PCR) TBE (Ab)	Dirofilaria immitis (antigen) Leishmania (Ab) Babesia canis (Ab) Neospora caninum (Ab) Neospora caninum (PCR) Ehrlichia canis (Ab) TBE (Ab)
3	Yorkshire Terrier	f	58	No	–	–	–	No	No	No	No		Neospora caninum (Ab) Toxoplasma gondii (Ab)
4	Mixed breed	fs	47	No	–	–	–	No	No	No	No	Neospora caninum (PCR) Toxoplasma gondii (PCR) TBE (PCR and Ab) Lyme disease (PCR) Cryptococcus neoformans/C. gattii (PCR) Bacterial culture	Distemper virus (PCR) *Anaplasma* spp. (PCR) Babesia (PCR) Ehrlichia sp. (PCR) Hepatozoon canis (PCR) Bartonella sp. (PCR)
5	Boston Terrier	f	15	No	–	–	–	Yes	No	No	No	Neospora caninum (PCR) Toxoplasma gondii (PCR) Distemper virus (PCR)	
6	French Bulldog	m	55	Yes	58	2 mg/kg q24h (2 weeks 1 mg/kg q24h (4 weeks) 0.75 mg/kg q24h (4 Weeks) 0.5 mg/kg q24h (4 weeks) 0.5mg/kg q48h (4 weeks) 0.5 mg/kg q24h (4 weeks) Treated with 0.3 mg/kg q24h for 2 weeks when included into study	-	Yes	Yes	No	No		Neospora caninum (Ab) Toxoplasma gondii (Ab)
7	Mixed breed	mc	84	No	–	–	–	Yes	No	No	No	Neospora caninum (PCR) Toxoplasma gondii (PCR) Distemper virus (PCR)	
8	Pomeranian	m	83	No	–	–	–	Yes	No	No	No	Neospora caninum (PCR) Toxoplasma gondii (PCR) Distemper virus (PCR)	
9	Chihuahua	f	47	No	–	–	–	No	Yes	No	LEV 20 mg/kg q8h	Neospora caninum (PCR) Toxoplasma gondii (PCR) Distemper virus (PCR) TBE (Ab)	
10	Chihuahua	fs	108	No	–	–	–	No	No	No	No	Distemper virus (PCR)	Neospora caninum (Ab) Toxoplasma gondii (Ab)

Ab, antibodies; ASM, anti-seizure medication; f, female; fs, female spayed; m, male; mc, male castrated; MRI, magnetic resonance imaging; LEV, Levetiracetam; RT, radiation therapy; TBE, tick-borne encephalitis; CSF, cerebrospinal fluid.

Seven dogs were newly diagnosed with MUO, and three dogs (dog 1, 2, and 6) were diagnosed with a MUO relapse. These three dogs were treated with variable immunosuppressive medication before enrollment in the study. Further information is given in Table 1. For the study purpose, immunomodulating medication other than prednisolone was stopped before inclusion.

### 3.2. Pre-treatment neurodisability score, MRI, and CSF findings

**Figure 1:**
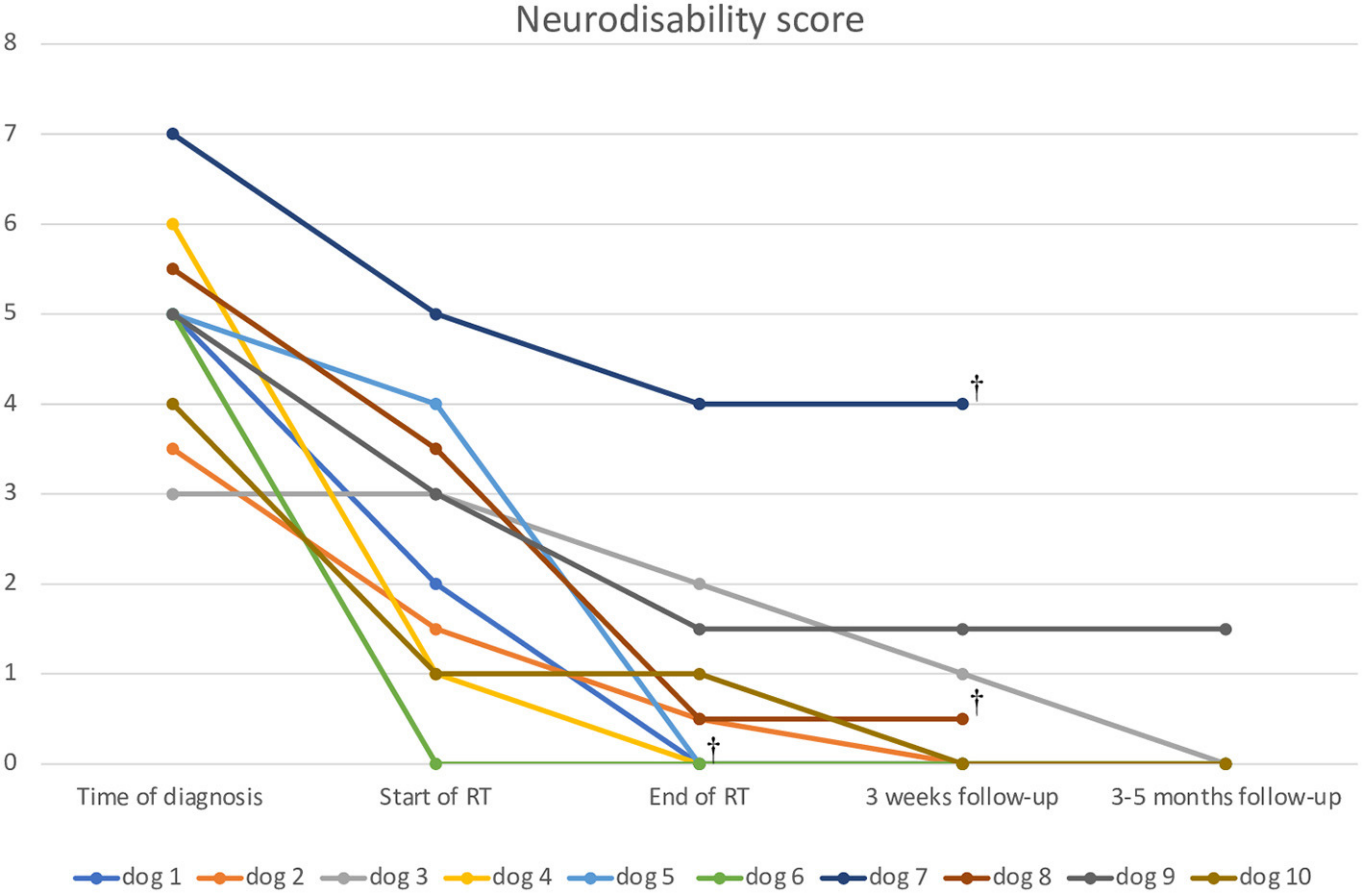
The line diagram shows the progression of the neurodisability score of the dogs examined. The x-axis shows the time course, and the y-axis shows the disability score at the time of the examination.

General clinical examination and blood results were unremarkable in all dogs. The neurodisability score varied among the study population and ranged between 3 and 7 (mean 4.9) prior to radiation therapy (Figure 1).

MRI revealed focal lesions in two and multifocal lesions in eight dogs. After contrast admission, enhancement was observed in all cases with variable pattern. Mild to moderate mass effect was reported in six dogs. We recognized four dogs with a mild foramen magnum herniation. One dog did have an additional mild caudal transtentorial herniation. Further details on MRI characteristics including imaging based subclassification into GME, NLE, and NME are reported in Table 2. We identified two cases of suspected NLE, which shared also features of GME due to the involvement of cerebral and cerebellar cortical gray matter.

**Table 2:** MRI and CSF findings.

**Case no**.	**MRI before RT**					**CSF before RT**		**Follow-up MRI after RT**						**CSF after RT**	
	**Lesion pattern**	**Affected areas**	**Contrast pattern**	**Mass effect and herniation**	**MUO subclassification based on MRI**	**TNCC (WBC/μL)**	**TP (g/L)**	**Time between RT and MRI (months)**	**Course**	**Lesion pattern**	**Affected areas**	**Contrast pattern**	**Mass effect and herniation**	**TNCC (WBC/μL)**	**TP (g/L)**
1	Multifocal	Predominantly subcortical white, in less extend also cortical gray matter; temporal and parietal regions of both cerebral hemispheres; thalamus bilateral; left pons; right medulla oblongata	Multifocal, heterogeneous, mild CE in affected brain parenchyma	No mass effect by lesions; no herniation	NLE with possible overlap with GME due to involvement of cortical gray matter	0.7	0.25				–	–	–	–	–
2	Multifocal	Cortical gray and subcortical matter; frontal and temporal regions of both cerebral hemispheres	Multifocal, heterogeneous, mainly strong CE in affected brain parenchyma and associated meninges	Moderate mass effect in frontal region on falx cerebri and right cerebral hemisphere as well as on both lateral ventricles; no herniation	GME	11	0.46	5	Lesions partially resolved	Multifocal	Predominantly subcortical white, in less extend in less extend also cortical gray matter; frontal and temporal regions of both cerebral hemispheres	No CE	No mass effect; no herniation	2	0.32
3	Multifocal	Medulla oblongata (mainly right side) and left internal capsule	Focal, heterogeneous, moderate CE in right medulla oblongata	Minimal mass effect on falx cerebri and left cerebral hemisphere; no herniation	NLE	Not enough material for TNCC; lymphocytic pleocytosis	Not enough material for TP	5	Lesion in medulla oblongata partially resolved; lesions in left internal capsule progressed; new lesions in right internal capsule	Multifocal	Medulla oblongata (only right side), left and right internal capsule	No CE	Minimal mass effect on falx cerebri and left cerebral hemisphere; no herniation	8.7	0.46
4	Multifocal	Cerebellar and cerebral cortical gray and subcortical white matter, both hemispheres	Focal, peripheral, moderate CE left cerebellar hemisphere and associated meninges	No mass effect; no herniation	GME	349.7	Pandy‘s reaction +++	3	Complete resolution	Complete resolution	Complete resolution	No CE	No mass effect; no herniation	1.3	0.17
5	Multifocal	Predominantly cerebral subcortical white matter; left and right frontal and left temporal regions; left thalamus; right pons	Multifocal, heterogeneous, moderate CE in affected brain parenchyma; in subcortical white matter of left cerebral hemisphere with peripheral/ring-like CE	Mild mass effect on falx cerebri and left right cerebral hemisphere; mild cerebellar foramen magnum herniation	NLE	36.6	0.3	3	Lesions partially resolved	Multifocal	Cerebral subcortical white matter; left and right frontal and left temporal region	No CE	No mass effect; no herniation	7.7	0.32
6	Multifocal	Cortical gray and subcortical white matter of cerebellum and left cerebral hemisphere	Multifocal, heterogeneous, moderate CE of affected cerebellar parenchyma	Mild mass effect on 4th ventricle; no herniation	NLE with possible overlap with GME due to involvement of cerebellar gray matter	21	0.32	3	Lesions partially resolved	Multifocal	Cerebellar cortical gray and white matter	Multifocal, heterogeneous, moderate CE of affected cerebellar parenchyma	No mass effect; no herniation	31	0.32
7	Focal	Right thalamus, right and left mesencephalon and pons	Multifocal, heterogeneous, moderate CE in affected brain parenchyma	Mild mass effect on interthalamic adhesion and rostral aspect of cerebellum; mild caudal transtentorial and cerebellar foramen magnum herniation	GME	32	0.3								
8	Multifocal	Subcortical white matter; frontal, temporal, parietal and occipital regions of both cerebral hemispheres	Multifocal, heterogeneous, strong CE of affected brain parenchyma	No mass effect; no herniation	NLE	2.8	0.2	2	Static lesions	Multifocal	Subcortical white matter; frontal, temporal, parietal and occipital regions of both cerebral hemispheres	No parenchymal CE, mildly increased meningeal CE both cerebral hemispheres	No mass effect; no herniation	6.8	0.26
9	Focal	Predominantly subcortical white, in less extend also cortical gray matter; frontal and temporal regions of left cerebral hemisphere; thalamus and caudate nucleus left cerebral hemisphere	Predominantly peripheral, heterogeneous, strong CE in affected brain parenchyma	Moderate mass effect on left cerebral hemisphere; mild cerebellar foramen magnum herniation	NME	7.3	0.33	3	Lesions partially resolved	Focal	Predominantly subcortical white, in less extend also cortical gray matter; frontal and temporal regions of left cerebral hemisphere; thalamus and caudate nucleus left cerebral hemisphere	No CE	No mass effect; no herniation	6	0.17
10	Multifocal	Lesions distributed bilateral, asymmetric over all 5 brain divisions; affecting both, gray and white matter	Multifocal, heterogeneous, mild CE in affected brain parenchyma	No mass effect; mild tipping of cerebellar vermis into foramen magnum	GME	542.6	1.53	3	Complete resolution	Complete resolution	Complete resolution	No CE	No mass effect; mild tipping of cerebellar vermis into foramen magnum	3.7	0.23

For cases that were included with a relapse into the study, the last MRI before beginning radiation therapy is described.

CE, contrast enhancement; GME, granulomatous meningoencephalitis; MRI, magnetic resonance imaging; NLE, necrotizing leukoencephalitis; NME, necrotizing meningoencephalitis; RT, radiation therapy, TNCC, total nucleated cell count; TP, total protein; CSF, cerebrospinal fluid.

The CSF total nucleated cell count ranged between 0.7 and 542.6 leukocytes per μL. In two dogs (dog 1 and 8) the cell count was normal. In both, MUO was later confirmed by histopathology. In one dog only a small amount of CSF could be collected, precluding counting of the exact cell count. However, a lymphocytic pleocytosis was confirmed by a board-certified clinical pathologist. CSF total protein count varied between 0.2 and 1.53 g/L, with normal protein levels in four dogs. In one dog CSF total protein was not measured due to low CSF volume obtained. In another dog total protein was semiquantitively determined by Pandy‘s test. The test was rated strongly positive (+++). For further details on CSF results, the reader is referred to Table 2.

Infectious diseases were tested negative in all dogs. Infectious disease testing varied among the patients depending on additional clinical symptoms, season, traveling history and availability of body fluids. For further details on the individual infectious disease testing the reader is referred to Table 1.

### 3.3. Radiation therapy

In the seven dogs with newly diagnosed MUO radiation therapy was initiated after a mean of 9.3 days (SD 3.6 days; range 6–16 days). In the remaining three dogs, time to relapse was 474, 802, and 111 days, respectively, and RT was started after a mean of 26 days (SD 23.5 days; range 6–52 days) after confirmation of relapse. In the time between MUO diagnosis and beginning of radiation therapy all dogs were treated with prednisolone monotherapy. After infectious diseases were ruled out, dogs were treated orally with 2 mg/kg prednisolone every 24 h. The dose was reduced after two weeks to 1 mg/kg per day. Further reductions were made approximately every six weeks by 25% in order to achieve the lowest effective dose.

In all dogs the whole brain was considered as radiation target (mean volume 68.5 cm^3^, ±SD 15.8 cm^3^, range 50.7–93.2 cm^3^). The near-maximum dose (D_2%_) to the PTV was 20.5 Gy (mean); ±SD 0.3 Gy; the median dose (D_50%_) to the PTV was 20 Gy (mean); ±SD 0 Gy and the near-minimum dose (D_98%_) to the PTV was 18.9 Gy (mean); ±SD 0.7 Gy). The mean treatment time was six days (range 5–7 days).

### 3.4. Follow-up and outcome

#### 3.4.1. Short term follow-up

The neurodisability score decreased in all but one dog between diagnosis and start of radiation therapy and further decreased during and after RT (Figure 1).

#### 3.4.2. Follow-up MRI and CSF

**Figure 2:**
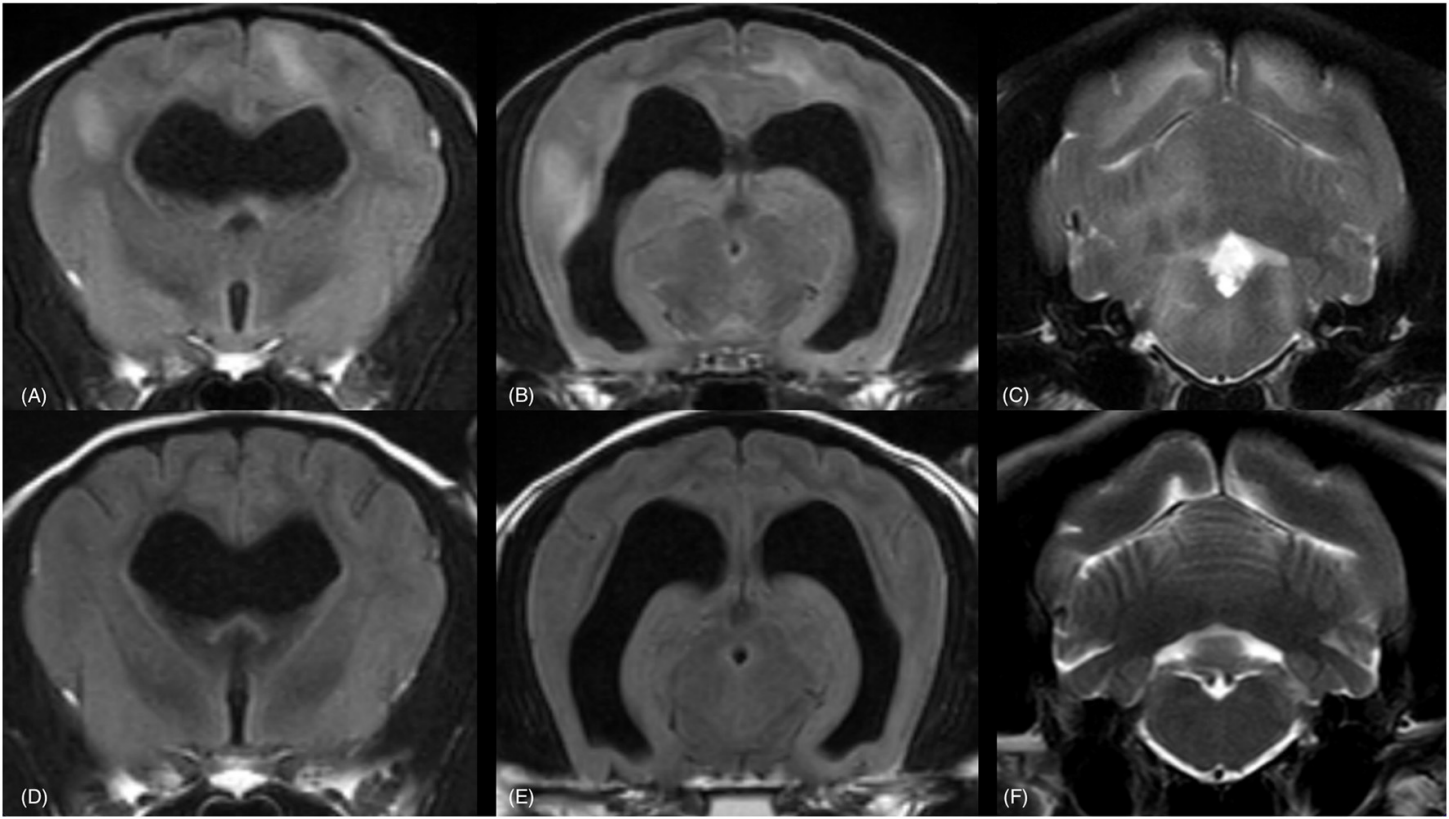
MRI of a 9-year-old Chihuahua (dog 10). Complete resolution of lesions is visible. In the upper row the brain is imaged at the time of diagnosis **(A–C)** and in the bottom row the brain is imaged at the corresponding levels three months after radiation therapy **(D–F)**. The sequences are from left to right in a rostro-caudal direction at the level of the optic chiasm **(A, D)**, the rostral colliculi and medial geniculate bodies **(B, E)** and the cerebellar nuclei **(C, F)**. Transverse FLAIR (TR = 11,000 ms, TE = 125 ms, TI = 2,800 ms, ST = 2.5 mm) **(A, B, D, E)** and T2w (TR = 5,774 ms, TE = 100 ms, ST = 2.5 mm) **(C, F)** images are provided. TR, Time of Repetition; TE, Time of Echo; TI, Time to Inversion; ST, Slice Thickness. Multifocal, bilateral, asymmetrical, poor defined, hyperintense lesions, mainly affecting the white matter are visible **(A–C)**. The lesions are in complete remission at the time of follow up—MRI **(D–F)**.

**Figure 3:**
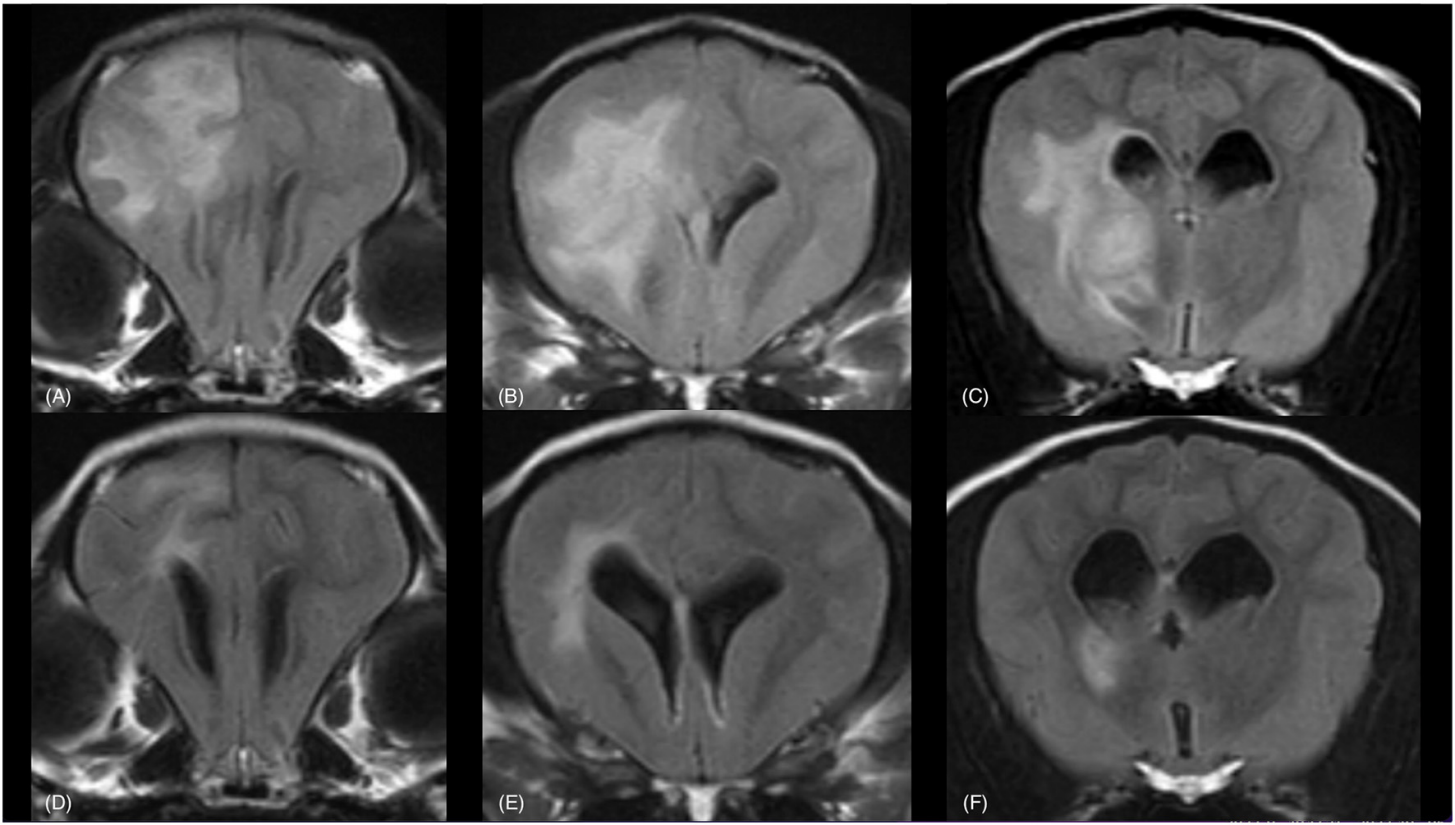
MRI of a 4-year-old Chihuahua (dog 9). Partial resolution of lesions is visible. In the upper row the brain is imaged at the time of diagnosis **(A–C)** and in the bottom row the brain is imaged at the corresponding levels three months after radiation therapy **(D–F)**. The sequences are from left to right in a rostro-caudal direction at the rostral commissure **(A, D)**, the lateral ventricles, caudate nucleus, internal capsule and body of the corpus callosum **(B, E)** and the optic chiasm **(C, F)**. Transverse FLAIR (TR = 11,000 ms, TE = 125 ms, TI = 2,800 ms, ST = 2.5 mm) **(A–F)** images are provided. TR, Time of Repetition; TE, Time of Echo; TI, Time to Inversion; ST, Slice Thickness. A large, single, irregular, and apparently well-delineated, hyperintense lesion within the left forebrain is visible, mainly affecting the white matter **(A–C)**. The lesions are partially resolved at the time of follow-up MRI **(D–F)**.

Follow-up MRI was performed in 8/10 dogs. Results of the follow-up MRI and CSF findings are reported in Table 2. MRI images demonstrating complete and partial resolution of the lesions are shown in Figures 2, 3, respectively. Follow-up CSF examination was performed in 8/10 cases.

Follow-up MRI and CSF examination was not performed in two dogs, because they did not reach the 3-month follow-up time. Follow-up MRI and CSF examination were not performed after three, but five months in two cases, due to restrictions related to the coronavirus pandemic.

#### 3.4.3. Outcome

At the time of writing 6/10 dogs are still alive. No dog was lost to follow-up. Median follow-up time for the dogs still alive was 1,033 days (95% CI: 28–2,029), mean 779 days (range 317–1,082 days).

**Figure 4:**
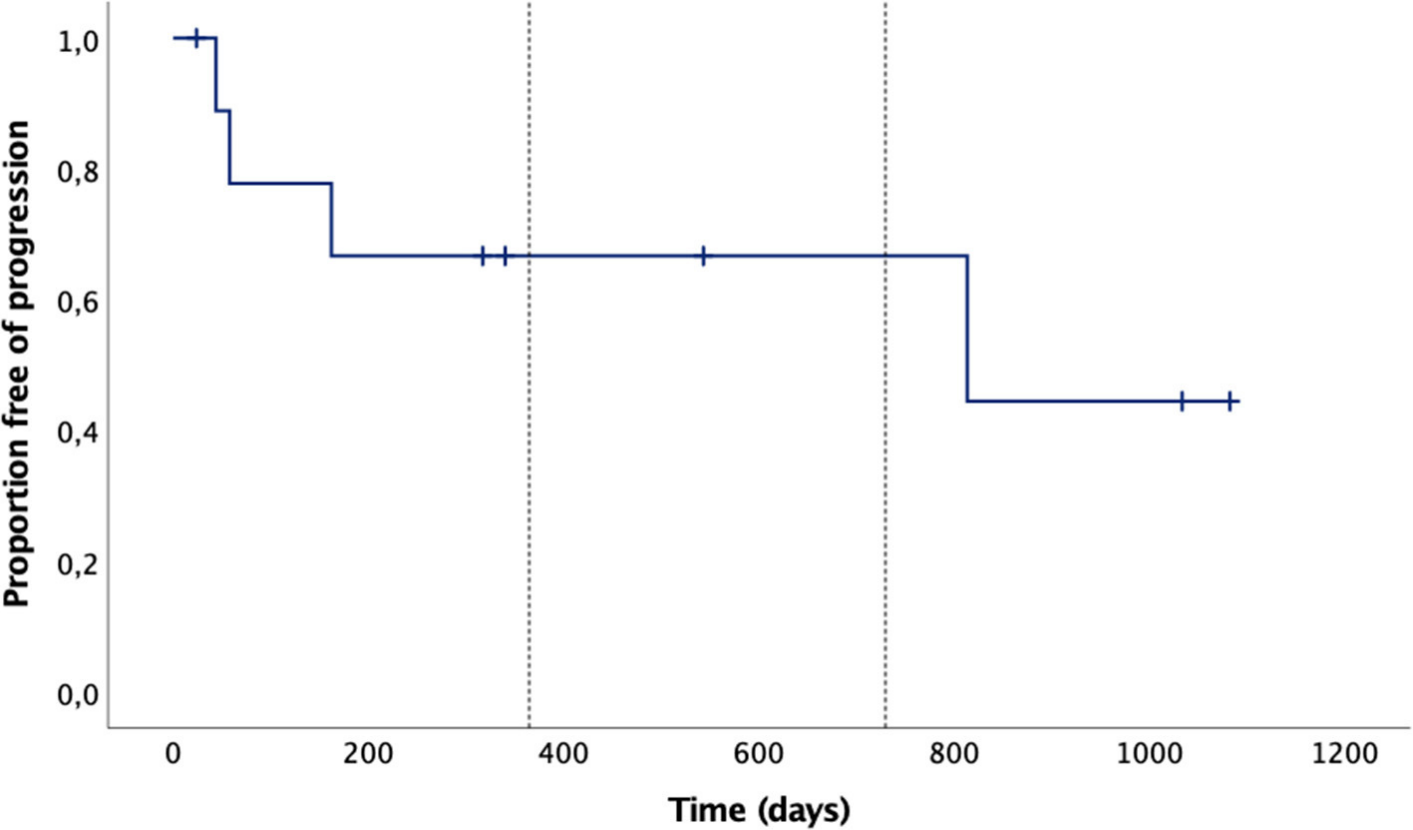
Time to progression Kaplan-Meier curves of the 10 dogs: mean time to progression was 691 days (95%CI: 396–987), median not reached. The tick marks represent censored cases, the vertical dotted lines mark 1 and 2 years.

**Figure 5:**
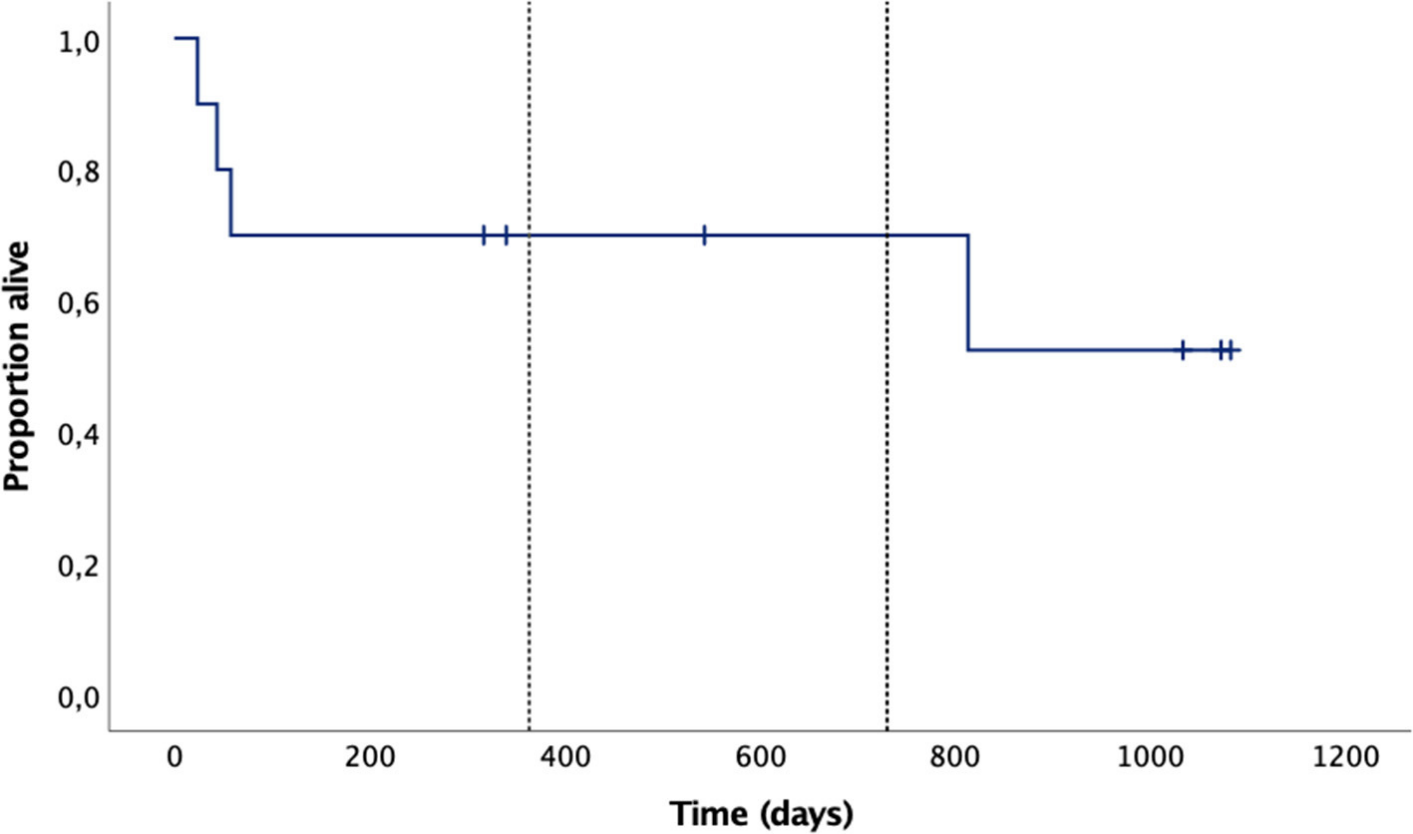
Overall survival Kaplan-Meier curves of the 10 dogs: mean overall survival was 723 days (95%CI: 436–1,011), median not reached. The tick marks represent censored cases, the vertical dotted lines mark 1 and 2 years.

Overall, four dogs showed progression: mean time to clinical progression for all dogs was 691 days (95%CI: 396–987), median not reached. Mean overall survival for all dogs was 723 days (95%CI: 436–1,011), again, median not reached. The Kaplan-Meier curves for the time to progression and the overall survival time are given in Figures 4, 5, respectively.

Clinical assessment as well as follow-up MRI and CSF examination were unremarkable in dog 4 and dog 10. At the time of writing, they are still in complete remission (1,033 and 340 days after beginning of radiotherapy, respectively), medicated with solely prednisolone at this point.

Five dogs were treated with a second immunosuppressive drug. In dog 3 with new lesions (disease progression) on MRI mycophenolate mofetil was started (11.8 mg/kg twice daily). In the four other dogs with incomplete remission (dog 2, 5, 6, and 9) ciclosporin was added. Dosages varied between 3.1 and 7.1 mg/kg twice daily.

Three dogs (dog 1, 8, and 5) were presented because of acute neurological deterioration 45, 59, and 814 days after beginning of radiation therapy. At this time treatment was made by prednisolone only in all dogs except dog 5, which was additionally treated with ciclosporin and cytosine-arabinoside. In all dogs relapse was clinically suspected or confirmed based on imaging criteria. The dogs were euthanized on owner's request. Relapse was histopathologically confirmed in all dogs.

Dog 7 was presented 25 days after the beginning of radiation therapy due to reduced general condition, apathy, weight loss, and severe polyuria and polydipsia. A (diabetic) ketoacidosis was diagnosed, and euthanasia of the dog performed based on the owner's request. Surprisingly, glioma was suspected based on histopathologic findings in necropsy.

### 3.5. Histopathology

Dog 1 was diagnosed with a chronic, moderate, and multifocal to diffuse and granulomatous meningoencephalitis. Perivascular cuffs were seen in multifocal areas of the cerebral cortex, subcortical white matter, and basal nuclei as well as in the cerebellum. Inflammatory cells were dominated by lymphocytes and macrophages. Immunohistochemistry was not performed.

Dog 8 was diagnosed with a chronic, high-grade, necrotizing encephalitis. Bilateral, asymmetric, necrotic lesions were seen within the cerebral white matter, transitioning into the thalamus. Inflammation was sparse and mild, dominated by macrophages. Interestingly no CD3+ T-lymphocytes were recognized by immunohistochemistry.

Dog 5 was diagnosed with a chronic, severe, and multifocal, necrotizing, lymphohistiocytic leukoencephalitis. Perivascular cuffs were found in the frontal cortex, internal capsule, corona radiata and among various white matter tracts of the brainstem. Inflammatory cells were dominated by lymphocytes and macrophages. Next to those lesions there were also focal-extensive, necrotic areas within the cerebral white matter containing few mononuclear cells. These areas matched the lesions seen on the first MRI examination and follow-ups. They were in line with chronic, burned-out lesions of necrotizing leukoencephalitis. In contrast, no specific changes suggestive for radiation associated side effects were identified.

No definitive diagnosis could be made for dog 7. Histopathological evaluation revealed a single, chronic, malacic lesion extending from the right thalamus to the pons with distinct astrocytic gliosis. Inflammatory infiltrates were not seen. Indeed, IHC was negative for CD3 and CD20. In the perivascular parenchyma only few Iba-1 positive macrophages were proven. In contrast, a mildly increased density of oligodendrocytes (positive for Olig2) was recognized in the periphery and within the lesion. The most likely diagnosis by the pathologist was an oligodendroglioma.

## 4. Discussion

We evaluated a small group of dogs with an imaging diagnosis of MUO that were treated with whole brain radiation and combined prednisolone therapy. All dogs in our study improved neurologically during the therapy, three dogs relapsed, and five dogs needed additional immunosuppressive treatment. Hence the effect of prednisolone and combined radiation therapy might be considered a temporary one.

Currently, only three studies including 17 dogs in total have evaluated the effect of brain radiation for MUE in conjunction to prednisolone treatment (13–15). In contrast to our currently used protocol, previous protocols were given with higher total doses and more, albeit smaller fractions. Muñana and Luttgen treated the lesions of dogs with higher (antitumor) total doses of 40–49.5 Gy, applied in 2.4–4.0 Gy fractions (14). In a previous study by our group on the other hand, the whole brain was irradiated with a classical whole-brain-irradiation protocol, as used to palliate brain metastasis in humans, with 10 fractions of 3 Gy (15). Although the radiotherapy protocols in the dogs treated differed, response rates were high and median survival times ranged between 404 and 476 days.

The rationale of using ionizing radiation for inflammatory conditions is based on the strong radiosensitivity of immune cells. Cells of the immune system often undergo apoptosis within 3–5 h of relatively low doses of radiation (25). In lymphoma treatment for example, total lymphoid irradiation to a dose of 30–40 Gy leads to a long-lasting T-cell depletion, which also can be used to treat autoimmune disease (26).

The exact pathophysiology of MUO remains unknown. However, the disease shares histopathological similarities with multiple sclerosis (MS) in people (27–29) and is characterized by lymphocyte infiltration of central nervous parenchyma. Especially T-cells are thought to play a major role (30), supported by elevated interferon-gamma (IFNγ) and interleukin-17 (IL17) mRNA levels and protein in brain tissue (31) and higher amount of IL-17 producing T-cells in the peripheral blood of affected dogs (32).

Immunohistochemistry was performed in all, but one dogs that underwent necropsy and histopathological evaluation within the first three months after radiotherapy. Interestingly, in dog 8 no CD3+ T-lymphocytes were observed. The lack this cell population might reflect the chronicity of the necrotizing lesions, but also could provide evidence for the radiation-induced apoptosis in lymphoid cells.

Despite incomplete understood pathophysiology, immunosuppressive agents are the mainstay of therapy (2, 4). Glucocorticoids, such as prednisolone, have proofed to be the most effective treatment. Based on a systematic review (11), the use of second line immunosuppressive agents is recommended as it may decreases glucocorticoid related side-effects (33) and increases treatment sufficiency. By doing so, multimodal treatment therapy has in the past shown to result in longer median survival time than glucocorticoid treatment alone (11, 12, 14, 34). However, recent studies did not show improved short-term survival in dogs treated with a second-line medication compared to sole prednisolone (6, 7). A reason for the variation of survival times among studies possibly reflects different immunosuppression protocols, with more aggressive and immunosuppressive glucocorticoid protocols may leading to longer survival times (33, 35).

The secondary immunosuppressive agents studied in MUO include antiproliferative agents (e.g., lomustine, procarbazine, cyclophosphamide), antimetabolites (e.g., cytosine arabinoside, azathioprine, mycophenolate mofetil, leflunomide) and calcineurin inhibitors (e.g., cyclosporine) (2, 4). Many of these more established medical second line immunosuppressive agents carry the risk of side effects, including gastrointestinal disturbances (36, 37), the potential risk for hepatotoxicity (38), myelosuppression (12) and more drug-specific side effects (3, 39, 40). Thus, alternative treatments for MUO are required.

In contrast, mild early radiation associated side effects of skin and hair were rarely reported by Beckmann et al. (15). Such side effects were neither noted in clinical follow-ups, nor reported by the owners in our study. This finding is in line with previous studies (14).

Four dogs in our study were euthanized, including three dogs within 59 days and one dog 814 days after beginning of radiotherapy. All dogs underwent histopathological examination of the brain. Histopathological features of radiation associated side effects were not noted in any of these dogs. For doses equivalent to 10 × 3 Gy, the commonly used protocol for whole-brain radiotherapy in people with brain metastasis, the risk of radionecrosis is very low, with an estimated risk of 0–1.6% (41, 42). This may provide further evidence for the safety of the described radiation protocol. Slow dividing and post-mitotic tissue such as the central nervous system, are more prone to late radiation side effects occurring from 6 months to years after radiation therapy. Such late side-effects are mainly attributed to injury of oligodendrocytes and endothelial cells, leading to white matter demyelination and vascular injury and depend on total dose, fraction size and target volume (43, 44). In human patients undergoing whole brain radiation therapy, vascular injury has been associated with cognitive decline (45, 46). In dogs a possible cognitive decline might not pose the same quality-of-life issues as in people. Though no side effects have been identified, we must admit that only one of the dogs that underwent histopathological evaluation was euthanized after more than 6 months and together with the small total number of dogs included into the study, we are unable to claim definitive harmlessness of the protocol.

The neurodisability score improved in all our patients already after initiation of prednisolone therapy and further improved during and after radiation therapy.

Clinical follow-up as well as MRI and CSF examinations revealed that only two dogs underwent complete remission. In those dogs, therapy was continued by tapering the dose of prednisolone. In the other dogs established second line medications were added to the prednisolone treatment, including cyclosporine in four and mycophenolate mofetil in one dog. A reason for that finding might be the fact, that radiation therapy was applied to the brain only. As stated above, this leads to a fast killing of inflammatory cells within the brain parenchyma but does not necessarily suppress the recruitment of inflammatory cells from the periphery. In human medicine this problem has been faced by total lymphoid radiation using even lower radiation doses (47–49).

Despite few prognostic indicators have been identified for short-term survival, the overall clinical course of MUO is often progressive and unpredictable. In many affected dogs the response to standard immunosuppressive therapy might be temporary.

Independently from chosen treatment protocol, 26–33% of the affected dogs are reported to die within the first 3 months after diagnosis (3, 5–7). Similar to these results, 3/10 dogs died or were euthanized within 59 days after start of radiotherapy in our study. A recent publication identified obtundation at presentation as risk factor for early euthanasia in dogs with MUO (7). In line with this study, all three dogs that have been euthanized within the first 3 months after radiotherapy in our study, presented obtunded.

Steroid responsive meningitis arteritis (SRMA) is another immune-mediated, inflammatory disease in dogs. As in MUO, immunosuppression by prednisolone is considered the mainstay therapy (50). Few data on the management of relapsing SRMA cases is available, using medications in conjunction to prednisolone that are also described in MUO (50–52). One of these studies has shown, that increasing prednisolone dosage was not sufficient to control clinical signs in 40% of the cases (51). The reason for this finding remains unknown. However, reduced sensitivity to glocorticoids has been noticed in people as well, including patients with multiple sclerosis (53, 54).

In the lack of a standard treatment protocol, yet only a few treatment studies included dogs with relapsing MUO (55). Similar to SRMA, it‘s reasonable to conclude that relapsing MUO may require the combination of prednisolone with other immunosuppressive drugs.

Our results show that radiotherapy might be beneficial in dogs that are considered (partially) refractory to medical treatment alone. All three dogs, that were considered relapses showed a quick response to radiotherapy in combination with prednisolone, leading to a neurodisability score of 0 in two of them and 0.5 in one of them by the end of radiotherapy. This compares to preliminary findings were in 11/14 dogs refractory to medical treatment improvement was observed after RT (56). Since the specific effect of radiation on inflammation is not yet understood we do not know if the non-responding groups in medical and radiation treatment overlap. It may well be possible that dogs failing one treatment would respond to the other.

Recently a study highlighted, that early death might be related to medical side effects in ~20% of dogs that die or are euthanized (7). Similar to previous results (15), radiation associated side effects were neither noted in short, nor in long-term follow-up in our study. In contrast, glucocorticoid-induced diabetes mellitus was the cause for euthanasia in one dog in our study. Based on histopathological findings an oligodendroglioma has been diagnosed in this dog. As the histopathological diagnosis of immune mediated encephalitis was not part of our inclusion criteria, we still included the case in our results. The patient had the worst neurodisability score at the time of diagnosis and by the end of radiotherapy (7 and 4, respectively). The inclusion of the dog with suspected oligodendroglioma reflects the poorly understood etiopathogenesis of MUO, the lack of accurate, non-invasive antemortem diagnostic tests and, as in this case, effective treatment.

Based on previously published MR imaging features, we identified four dogs with suspected GME, five dogs with suspected NLE and one dog with suspected NME. Please note, that one of the dogs with GME was diagnosed with oligodendroglioma in pathology. Interestingly, two dogs fit predominantly the imaging features of NLE, but also shared features GME due to the involvement of cerebral and cortical gray matter (Table 2). Both dogs were French Bulldogs and have been diagnosed with relapsing MUO before included into our study (Table 1). One of these dogs underwent histopathologic examination of the brain, which also identified features of GME. Classically, NLE has been described in French Bulldogs (22, 57, 58). However, the overlap of necrotizing and granulomatous encephalitis has been noted recently, suggesting that the transitions between necrotizing and granulomatous variants of MUO might be flowing and less distinct in some cases (59).

A complete remission was achieved in two dogs, both with imaging features of GME. However, we believe that our study population is too small to draw definitive conclusions on the response to radiotherapy specified to the variants of MUO.

Our study population comprised two dogs suffering from epileptic seizures. The prognosis to achieve a good seizure outcome is generally considered poor in dogs with structural epilepsy (60), including MUO (61). Despite only one of the dogs in our study had anticonvulsive medication (Levetiracetam 20 mg/kg q8h), both became seizure free. The reasoning for the treatment with Levetiracetam was made on previous investigations, highlighting a slightly beneficial outcome when used in structural epilepsy (62). Although our study sample is too small to draw definitive conclusions, radiotherapy may also have contributed to this outcome. Significantly longer duration of seizure-freedom has been observed in dogs with brain tumors undergoing radiotherapy compared to medical treatment alone (63). However, the reason for this finding remained unclear.

Our study has several limitations. First, this study was designed in a small scale and without a control group receiving sole prednisolone or in combination with a second immunosuppressive drug. Second, radiotherapy was not performed alone, but in conjunction to prednisolone therapy. For these reasons, the entire benefit of RT cannot be quantified in our study. However, due to the fast improvement of the neurodisability score in conjunction with improved lesion pattern seen on MRI in most of our dogs, we assume a complementary therapeutic effect on immunosuppressive medication as it has been also reported in previous publications (14, 15). Another limitation is the lack of histopathological confirmation at inclusion. In one dog oligodendroglioma was suspected based on post-mortem histopathological findings.

The histopathological suspicion of an oligodendroglioma in one of our cases shows, that there still is a risk to include different CNS diseases like neoplasia.

Comparative prospective studies are needed to better understand the value of radiotherapy for the treatment of MUO vs. drug treatment options. To proof the potential benefit of second line medication in overcoming glucocorticoid associated side effects, future treatment protocols (using chemotherapeutics like cytarabine) should include scoring systems (64).

In conclusion, our results support that radiotherapy may serve as fast, additional treatment option in conjunction to glucocorticoid treatment for dogs with meningoencephalitis of unknown origin without overt safety issues. It could be considered in dogs being refractory to medical treatment alone or suffering from severe medical side effects.

## Data availability statement

The original contributions presented in the study are included in the article/supplementary material, further inquiries can be directed to the corresponding author.

## Ethics statement

The animal study was reviewed and approved by Animal Ethics Council of the Canton of Zurich, Switzerland. Written informed consent was obtained from the owners for the participation of their animals in this study.

## Author contributions

RH was responsible for data collection regarding clinical, neurological, and laboratory data, their interpretation and drafting, and writing the manuscript. KB contributed to data collection and writing and helped to draft the manuscript. MK performed radiation therapy. FS contributed to conception, design and data collection, and gave critical input. Furthermore, he was involved to raise funding of the study. CR performed radiation therapy and contributed to conception, design, and writing and finalized the version to be published. Furthermore, she was involved to raise funding of the study. All authors have read and approved the final version of the manuscript.

## References

[1] Coates JR Barone G Dewey CW Vitale CL Holloway-Azene NM Sessions JK. Procarbazine as adjunctive therapy for treatment of dogs with presumptive antemortem diagnosis of granulomatous meningoencephalomyelitis: 21 cases (1998-2004). J Vet Intern Med. (2007) 21:100–6. 10.1111/j.1939-1676.2007.tb02934.x 17338156

[2] Coates JR Jeffery ND. Perspectives on meningoencephalomyelitis of unknown origin. Vet Clin North Am Small Anim Pract. (2014) 44:1157–85. 10.1016/j.cvsm.2014.07.009 25239815

[3] Smith PM Stalin CE Shaw D Granger N Jeffery ND. Comparison of two regimens for the treatment of meningoencephalomyelitis of unknown etiology. J Vet Intern Med. (2009) 23:520–6. 10.1111/j.1939-1676.2009.0299.x 19645837

[4] Cornelis I van Ham L Gielen I de Decker S Bhatti SFM. Clinical presentation, diagnostic findings, prognostic factors, treatment and outcome in dogs with meningoencephalomyelitis of unknown origin: a review. Vet J. (2019) 244:37–44. 10.1016/j.tvjl.2018.12.007 30825893

[5] Lowrie M Smith PM Garosi L. Meningoencephalitis of unknown origin: investigation of prognostic factors and outcome using a standard treatment protocol. Vet Rec. (2013) 172:527. 10.1136/vr.101431 23462382

[6] Cornelis I Volk HA van Ham L de Decker S. Prognostic factors for 1-week survival in dogs diagnosed with meningoencephalitis of unknown aetiology. Vet J. (2016) 214:91–5. 10.1016/j.tvjl.2016.05.008 27387733

[7] Lawn RW Harcourt-Brown TR. Risk factors for early death or euthanasia within 100 days of diagnosis in dogs with meningoencephalitis of unknown origin. Vet J. (2022) 287:105884. 10.1016/j.tvjl.2022.105884 35987308

[8] Schwab S Herden C Seeliger F Papaioannou N Psalla D Polizopulou Z Baumgärtner W. Non-suppurative Meningoencephalitis of Unknown Origin in Cats and Dogs: an Immunohistochemical Study. J Comp Pathol. (2007) 136:96–110. 10.1016/j.jcpa.2006.11.006 17275833 PMC7126569

[9] Kipar A Baumgärtner W Vogl C Gaedke K Wellman M. Immunohistochemical Characterization of Inflammatory Cells in Brains of Dogs with Granulomatous Meningoencephalitis. Vet Pathol. (1998) 35:43–52. 10.1177/030098589803500104 9545134

[10] Barnes Heller HL Granick MN Pinkerton ME Keuler NS. Case-control study of risk factors for granulomatous meningoencephalomyelitis in dogs. J Am Vet Med Assoc. (2019) 254:822–5. 10.2460/javma.254.7.822 30888272

[11] Granger N Smith PM Jeffery ND. Clinical findings and treatment of non-infectious meningoencephalomyelitis in dogs: A systematic review of 457 published cases from 1962 to 2008. Vet J. (2010) 184:290–7. 10.1016/j.tvjl.2009.03.031 19410487

[12] Flegel T Boettcher IC Matiasek K Oevermann A Doherr MG Oechtering G. Comparison of oral administration of lomustine and prednisolone or necrotizing encephalitis in dogs. J Am Vet Med Assoc. (2011) 238:337–45. 10.2460/javma.238.3.337 21281217

[13] Sisson AF LeCouteur RA Dow SW Gilette EL. Radiation therapy of granulomatous meningoencephalomyelitis of dogs. ACVIM forum proceedings. J Vet Intern Med. (1989) 3:119.

[14] Muñana KR Luttgen PJ. Prognostic factors for dogs with granulomatous meningoencephalomyelitis: 42 cases (1982-1996). J Am Vet Med Assoc. (1998) 212:1902–6. 9638190

[15] Beckmann K Carrera I Steffen F Golini L Kircher PR Schneider U. A newly designed radiation therapy protocol in combination with prednisolone as treatment for meningoencephalitis of unknown origin in dogs: a prospective pilot study introducing magnetic resonance spectroscopy as monitor tool. Acta Vet Scand. (2015) 57:4. 10.1186/s13028-015-0093-3 25637270 PMC4316757

[16] Talarico LR Schatzberg SJ. Idiopathic granulomatous and necrotising inflammatory disorders of the canine central nervous system: a review and future perspectives. J Small Anim Pract. (2010) 51:138–49. 10.1111/j.1748-5827.2009.00823.x 19814766

[17] Higgins RJ Dickinson PJ Kube SA Moore PF Couto SS Vernau KM. Necrotizing meningoencephalitis in five Chihuahua dogs. Vet Pathol. (2008) 45:336–46. 10.1354/vp.45-3-336 18487490

[18] Cherubini GB Platt SR Howson S Baines E Brodbelt DC Dennis R. Comparison of magnetic resonance imaging sequences in dogs with multi-focal intracranial disease. J Small Anim Pract. (2008) 49:634–40. 10.1111/j.1748-5827.2008.00628.x 18684139

[19] Cherubini GB Platt SR Anderson TJ Rusbridge C Lorenzo V Mantis P. Characteristics of magnetic resonance images of granulomatous meningoencephalomyelitis in 11 dogs. Vet Rec. (2006) 159:110–5. 10.1136/vr.159.4.110 16861389

[20] Flegel T Henke D Boettcher IC Aupperle H Oechtering G Matiasek K. Magnetic resonance imaging findings in histologically confirmed pug dog encephalitis. Vet Radiol Ultrasound. (2008) 49:419–24. 10.1111/j.1740-8261.2008.00400.x 18833947

[21] von Praun F Matiasek K Grevel V Alef M Flegel T. Magnetic resonance imaging and pathologic findings associated with necrotizing encephalitis in two Yorkshire Terriers. Vet Radiol Ultrasound. (2006) 47:260–4. 10.1111/j.1740-8261.2006.00137.x 16700176

[22] Flegel T. Breed-specific magnetic resonance imaging characteristics of necrotizing encephalitis in dogs. Front Vet Sci. (2017) 4:203 10.3389/fvets.2017.00203 29255715 PMC5723069

[23] Lotti D Capucchio MT Gaidolfi E Merlo M. Necrotizing encephalitis in a Yorkshire Terrier: clinical, imaging, and pathologic findings. Vet Radiology Ultrasound. (1999) 40:622–6. 10.1111/j.1740-8261.1999.tb00889.x 10608690

[24] Young BD Levine JM Fosgate GT de Lahunta A Flegel T Matiasek K. Magnetic resonance imaging characteristics of necrotizing meningoencephalitis in pug dogs. J Vet Intern Med. (2009) 23:527–35. 10.1111/j.1939-1676.2009.0306.x 19645838

[25] Hall EJ Giaccia AJ. Model tumor systems. IN: Radiobiology for the Radiologist. Philadelphia, PA: Wolters Kluwer (2019). p. 381.

[26] Hall EJ Giaccia AJ. Clinical response of normal tissues. In: Radiobiology for the Radiologist. Philadelphia, PA: Wolters Kluwer (2019). p. 363.

[27] Greer KA Wong AK Liu H Famula TR Pedersen NC Ruhe A. Necrotizing meningoencephalitis of Pug Dogs associates with dog leukocyte antigen class II and resembles acute variant forms of multiple sclerosis. Tissue Antigens. (2010) 76:110–8. 10.1111/j.1399-0039.2010.01484.x 20403140

[28] Prümmer J Stein V Marti E Lutterotti A Buch T Maiolini A. Oligoklonale Banden bei Hunden mit “meningoencephalitis of unknown origin” (MUO). Tierarztl Prax Ausg K Kleintiere Heimtiere. (2021) 49:75. 10.1055/s-0040-17224115

[29] Church ME Ceja G McGeehan M Miller MC Farias P Sánchez MD. Meningeal B cell clusters correlate with submeningeal pathology in a natural model of multiple sclerosis. J Immunol. (2021) 207:44–54. 10.4049/jimmunol.2000514 34162727 PMC8695639

[30] Uchida K Park E Tsuboi M Chambers JK Nakayama H. Pathological and immunological features of canine necrotising meningoencephalitis and granulomatous meningoencephalitis. Vet J. (2016) 213:72–7. 10.1016/j.tvjl.2016.05.002 27240919

[31] Park E-S Uchida K Nakayama H. Th1-, Th2-, and Th17-related cytokine and chemokine receptor mRNA and protein expression in the brain tissues, T cells, and macrophages of dogs with necrotizing and granulomatous meningoencephalitis. Vet Pathol. (2013) 50:1127–34. 10.1177/0300985813488957 23651736

[32] Barber R Barber J. Differential T-cell responses in dogs with meningoencephalomyelitis of unknown origin compared to healthy controls. Front Vet. Sci. (2022) 9:925770. 10.3389/fvets.2022.925770 35990273 PMC9386037

[33] Cornelis I van Ham L de Decker S Kromhout K Goethals K Gielen I. Sole prednisolone therapy in canine meningoencephalitis of unknown etiology. Vlaams Diergeneeskd Tijdschr. (2017) 86:24–8. 10.21825/vdt.v86i1.16300

[34] Jung DI Kang BT Park C Yoo JH Gu SH Jeon HW. A comparison of combination therapy (cyclosporine plus prednisolone) with sole prednisolone therapy in 7 dogs with necrotizing meningoencephalitis. J Vet Med Sci. (2007) 69:1303–6. 10.1292/jvms.69.1303 18176031

[35] Mercier M Barnes Heller HL. Efficacy of glucocorticoid monotherapy for treatment of canine meningoencephalomyelitis of unknown etiology: a prospective study in 16 dogs. J Vet Med Sci. (2015) 1:16–22. 10.1002/vms3.4 29067170 PMC5645807

[36] Barnoon I Shamir MH Aroch I Bdolah-Abram T Srugo I Konstantin L. Retrospective evaluation of combined mycophenolate mofetil and prednisone treatment for meningoencephalomyelitis of unknown etiology in dogs: 25 cases (2005-2011). J Vet Emerg Crit Care. (2016) 26:116–24. 10.1111/vec.12399 26458162

[37] Song J-H Yu D-H Lee H-C Hwang T-S Kim YJ An S-J. Evaluation of treatment with a combination of mycophenolate mofetil and prednisolone in dogs with meningoencephalomyelitis of unknown etiology: a retrospective study of 86 cases (2009–2017). BMC Vet Res. (2020) 16:192. 10.1186/s12917-020-02414-3 32532259 PMC7291637

[38] Wallisch K Trepanier LA. Incidence, timing, and risk factors of azathioprine hepatotoxicosis in dogs. J Vet Intern Med. (2015) 29:513–8. 10.1111/jvim.12543 25641386 PMC4895519

[39] Viviano KR. Glucocorticoids, cyclosporine, azathioprine, chlorambucil, and mycophenolate in dogs and cats. Vet Clin North Am Small Anim Pract. (2022) 52:797–817. 10.1016/j.cvsm.2022.01.009 35379498

[40] Hart SK Waddell L. Suspected drug-induced infiltrative lung disease culminating in acute respiratory failure in a dog treated with cytarabine and prednisone. J Vet Emerg Crit Care. (2016) 26:844–50. 10.1111/vec.12470 27062671

[41] Gondi V Bauman G Bradfield L Burri SH Cabrera AR Cunningham DA. Radiation therapy for brain metastases: an ASTRO clinical practice guideline. Pract Radiat Oncol. (2022) 12:265–82. 10.1016/j.prro.2022.02.003 35534352

[42] Raman S Mou B Hsu F Valev B Cheung A Vallières I. Whole brain radiotherapy versus stereotactic radiosurgery in poor-prognosis patients with one to 10 brain metastases: a randomised feasibility study. Clin Oncol. (2020) 32:442–51. 10.1016/j.clon.2020.02.001 32085923

[43] Harris D King GK Bergman PJ. Radiation therapy toxicities. Vet Clin North Am Small Anim Pract. (1997) 27:37–46. 10.1016/S0195-5616(97)50004-0 9002165

[44] Schultheiss TE Kun LE Ang KK Stephens LC. Radiation response of the central nervous system. Int J Radiat Oncol Biology Phys. (1995) 31:1093–112. 10.1016/0360-3016(94)00655-5 7677836

[45] Li J Bentzen SM Li J Renschler M Mehta MP. Relationship between neurocognitive function and quality of life after whole-brain radiotherapy in patients with brain metastasis. Int J Radiat Oncol Biol Phys. (2008) 71:64–70. 10.1016/j.ijrobp.2007.09.059 18406884

[46] Greene-Schloesser D Robbins ME Peiffer AM Shaw EG Wheeler KT Chan MD. Radiation-induced brain injury: a review. Front Oncol. (2012) 2:73. 10.3389/fonc.2012.00073 22833841 PMC3400082

[47] Devereux CK Vidaver R Hafstein MP Zito G Troiano R Dowling PC. Total lymphoid irradiation for multiple sclerosis. Int J Radiat Oncol Biol Phys. (1988) 14:197–203. 10.1016/0360-3016(88)90068-5 3275601

[48] Troiano R Devereux C Oleske J Denny T Hafstein M Zito G. T cell subsets and disease progression after total lymphoid irradiation in chronic progressive multiple sclerosis. J Neurol Neurosurg Psychiatry. (1988) 51:980–3. 10.1136/jnnp.51.7.980 2974471 PMC1033204

[49] Rohowsky-Kochan C Molinaro D Devereux C Troiano R Bansil S Zito G. The effect of total lymphoid irradiation and low-dose steroids on T lymphocyte populations in multiple sclerosis: correlation with clinical and MRI status. J Neurol Sci. (1997) 152:182–92. 10.1016/S0022-510X(97)00156-1 9415540

[50] Tipold A Schatzberg SJ. An update on steroid responsive meningitis-arteritis. J Small Anim Pract. (2010) 51:150–4. 10.1111/j.1748-5827.2009.00848.x 20070497

[51] Cizinauskas S Jaggy A Tipold A. Long-term treatment of dogs with steroid-responsive meningitis-arteritis: clinical, laboratory and therapeutic results. J Small Anim Pract. (2000) 41:295–301. 10.1111/j.1748-5827.2000.tb03205.x 10976624

[52] Günther C Steffen F Alder DS Beatrice L Geigy C Beckmann K. Evaluating the use of cytosine arabinoside for treatment for recurrent canine steroid-responsive meningitis-arteritis. Vet Rec. (2020) 187:e7. 10.1136/vr.105683 33638531 PMC7456679

[53] van Winsen LML Muris DFR Polman CH Dijkstra CD van den Berg TK Uitdehaag BMJ. Sensitivity to glucocorticoids is decreased in relapsing remitting multiple sclerosis. J Clin Endocrinol Metab. (2005) 90:734–40. 10.1210/jc.2004-0306 15546910

[54] Matysiak M Makosa B Walczak A Selmaj K. Patients with multiple sclerosis resisted to glucocorticoid therapy: abnormal expression of heat-shock protein 90 in glucocorticoid receptor complex. Mult Scler. (2008) 14:919–26. 10.1177/1352458508090666 18573821

[55] Zeira O Asiag N Aralla M Ghezzi E Pettinari L Martinelli L. Adult autologous mesenchymal stem cells for the treatment of suspected non-infectious inflammatory diseases of the canine central nervous system: safety, feasibility and preliminary clinical findings. J Neuroinflamm. (2015) 12:181. 10.1186/s12974-015-0402-9 26415563 PMC4587680

[56] Körner M Beckmann K Meier V Günther C Alisauskaite N Rohrer Bley C. Is radiation therapy a useful treatment option for meningoencephalomyelitis of unknown origin in dogs? In: ESVONC Congress Proceedings. Frankfurt: Nightingale Press Ltd. (2019). p. 47.

[57] Timmann D Konar M Howard J Vandevelde M. Necrotising encephalitis in a French bulldog. J Small Anim Pract. (2007) 48:339–42. 10.1111/j.1748-5827.2006.00239.x 17425701

[58] Spitzbarth I Schenk HC Tipold A Beineke A. Immunohistochemical characterization of inflammatory and glial responses in a case of necrotizing leucoencephalitis in a french bulldog. J Comp Pathol. (2010) 142:235–41. 10.1016/j.jcpa.2009.08.158 19815229

[59] Nessler JN Oevermann A Schawacht M Gerhauser I Spitzbarth I Bittermann S. Concomitant necrotizing encephalitis and granulomatous meningoencephalitis in four toy breed dogs. Front Vet Sci. (2022) 9:957285. 10.3389/fvets.2022.957285 36118343 PMC9477003

[60] Norona FE Volk HA. Investigating the efficacy of medical management for canine structural epilepsy. Vet Rec. (2020) 187:e63. 10.1136/vr.105708 32586969

[61] Kaczmarska A José-López R Czopowicz M Lazzerini K Leblond G Stalin C. Postencephalitic epilepsy in dogs with meningoencephalitis of unknown origin: clinical features, risk factors, and long-term outcome. J Vet Intern Med. (2020) 34:808–20. 10.1111/jvim.15687 31990104 PMC7096646

[62] Kelly D Raimondi F Shihab N. Levetiracetam monotherapy for treatment of structural epilepsy in dogs: 19 cases (2010-2015). Vet Rec. (2017) 181:1–7. 10.1136/vr.104190 28847876

[63] Monforte Monteiro SR Rossmeisl JH Russell J Holmes MA Wessmann A Morris J. Effect of radiotherapy on freedom from seizures in dogs with brain tumors. J Vet Intern Med. (2020) 34:821–7. 10.1111/jvim.15695 32032456 PMC7096644

[64] LeBlanc AK Atherton M Bentley RT Boudreau CE Burton JH Curran KM. Veterinary cooperative oncology group-common terminology criteria for adverse events (VCOG-CTCAE v2) following investigational therapy in dogs and cats. Vet Comp Oncol. (2021) 19:311–52. 10.1111/vco.12677 33427378 PMC8248125

